# Efficient design and implementation of approximate FA, FS, and FA/S circuits for nanocomputing in QCA

**DOI:** 10.1371/journal.pone.0310050

**Published:** 2024-09-06

**Authors:** Saeid Seyedi, Hatam Abdoli

**Affiliations:** Department of Computer Engineering, Faculty of Engineering, Bu-Ali Sina University, Hamedan, Iran; National University of Defense Technology, CHINA

## Abstract

Recently, there has been a lot of research in Quantum Cellular Automata (*QCA*) technology because it promises low power consumption, low complexity, low latency, and compact space. Simultaneously, approximate arithmetic, a new paradigm in computing, streamlines the computational process and emerges as a low-power, high-performance design approach for arithmetic circuits. Furthermore, the XOR gate has been widely used in digital design and is a basic building block that can be used in many upcoming technologies. The full adder (*FA*) circuit is a key component of QCA technology and is utilized in arithmetic logic operations including subtraction, multiplication, and division. A great deal of research has been done on the design of approximate FA, full subtractor (*FS*), full adder/subtractor (*FA/S*), and *4-bit* ripple carry adder (*RCA*) based on XOR logic, establishing them as essential components in the creation of QCA-based arithmetic circuits. This study presents three new and effective QCA-based circuits, based on XOR logic: an approximate FA, an approximate FS, an approximate FA/S, and an approximate *4-bit* ripple carry adder (*RCA*). Interestingly, some designs have inputs on one side and outputs on the other, making it easier to reach the components without being encircled by other cells and leading to a more effective circuit design. In particular, a delay of *0*.*5 clock phases*, an area of *0*.*01 μm^2^*, and implementation utilizing just *11* cells was accomplished in the approximate FA and subtractor designs. In a similar vein, the estimated FA/S designs showed *0*.*5 clock phase* delay, *0*.*01 μm^2^* area, and *12* cells used for implementation. An approximate *4-bit* RCA is proposed using *64* QCA cells. The effectiveness of these designs is evaluated through functional verification with the QCADesigner program. According to simulation results, these proposed solutions not only function well but significantly outperform previous ideas in terms of speed and space. The proposed FA, FS, and RCA designs surpassed the previous best designs by *21%*, *21%*, *and 43%*, respectively, in terms of cell count.

## 1. Introduction

Traditional, complementary metal oxide semiconductor technology has inherent problems regarding short-channel effects. There is a need to shift focus towards circuits based on quantum-dot cellular automata (*QCA*) to get around the shortcomings. QCA is a hot study subject because of its ultra-high computing speed and scalability of devices [1]. Additionally, the development of several multidisciplinary domains is aided by QCA technology [2]. This QCA technique has been the subject of several articles, with a particular emphasis on power consumption reduction and commercial feasibility [3]. The entire technology will need to be thoroughly improved over time to make sure that it is truly capable of quantum computing in the future [4].

From *67* transistors in *1965* to *16* billion in a device with a feature size of *5* nanometers, modern *CPUs* grew by Moore’s law [5]. However, since electrons can tunnel through gates smaller than *2* nanometers, the further increase in the device’s density leads to many technical problems, unwanted quantum effects, and high production costs. Also, reducing transistor sizes reduces the power required to drive them, generating less heat that becomes difficult to remove [6]. The net effect of this has been that considerable research is underway into alternative technologies that will allow scaling to continue. One such alternative for realizing nanoscale electrical devices comes through the development of QCA technology. Compared with traditional transistors, QCA circuits can be made more power-efficient, densely packed, and realized by conventional semiconductor manufacturing technologies. The binary digits *0 and 1* can be represented using QCA quantum dots in a regular grid [7]. The direction of quantum dots, which can be controlled by magnetic or electrical fields, determines the value passed through a circuit using quantum cascade architecture. A comparison between QCA and traditional silicon-based transistor technology yields several advantages. For example, QCA circuits can be much smaller and less power-hungry than transistor circuits. They are bound to revolutionize computers and other industries, mainly because they allow the production of very compact, more reliable, high-performance devices.

However, because the approximate computing paradigm stresses taking use of inherent flaws or tolerances in applications to obtain advantages in performance, power efficiency, and space utilization, it is crucial for circuit design. It is not the same as the traditional method of precise computation.

However, the inherent flaws or tolerances in applications are exploited much in the approximate computing paradigm to reap benefits in performance, power efficiency, and space; hence, it is really important in circuit design. That turns out not to be a traditional method of precise computation [8, 9]. This recognizes that most applications do not need exact results to perform their tasks, especially in signal processing, multimedia, machine learning, and nano-circuit designs. Rather, they will tolerate and even benefit from controlled errors or approximations. Huge power and hardware complexity benefits can be reaped if circuits and algorithms are allowed to be simpler because they were designed on the reduced requirement for exact computation. This comes particularly in handy in scenarios where the error resilience of an application is inherently built into the application and can sustain a more flexible and resource-efficient solution. Approximate circuits in hardware design give results sufficient for the application at hand but at lesser resources than their exact counterparts. Approximate circuits provide examples of approximate adders, multipliers, and memory units [10, 11].

Because they may be used to create devices that can process and analyze data, carry out a broad range of computational activities, and conduct sophisticated mathematical computations, full adder (*FA*), full subtractor (*FS*) circuits are essential components of digital circuits [12–14]. In this study, a new implementation of QCA-based approximate FA, FS, full adder/subtractor (*FA/S*), and *4-bit* RCA designs are proposed to minimize the number of cells, lowering the cost and complexity, and dissipating less energy and power.

The following is the order of the article flow. In Section 2, some of the principles of QCA technology have been explained. In addition, Section 2 reviews a succinct literature assessment of the adder and subtractor designs in QCA. Section 3 provides a detailed overview of the proposed QCA-based approximate FA, FS, FA/S, and 4-bit RCA architectures, while Section 4 provides a detailed analysis of the simulation results, design consumption, provides a detailed comparison and assessment of the QCA designs’ performance metrics with the cell, area and clock phases. The work provided is concluded in Section 5.

## 2. Background and related works

This section provides a brief description of the fundamental gates of QCA technology as well as the most recent and robust efforts on QCA FA and FSs.

### 2.1. QCA basics

**Fig 1:**
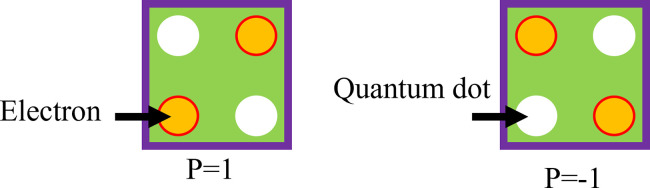
QCA cells.

**Fig 2:**
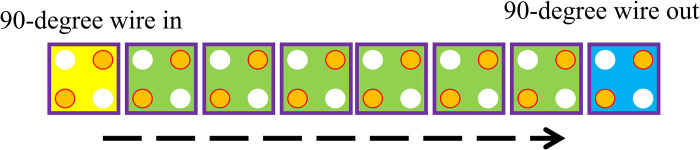
QCA wire by 90-degree cells.

A QCA cell, a square nanostructure filled with four quantum dots and two movable electrons that may travel between neighboring dots, is the fundamental part of QCA technology. QCA relies on the exact setting and interaction of cells, wherein the condition of each cell can be "flipped" according to the polarization of the cells neighboring it. It works on the principle of quantum tunneling effects and coulombic interactions. When transmitting signals, a line of QCA cells works by translating the state of one cell into the next through a domino effect. As such, the general organization of QCA cells is in grid form. In contrast, cells communicate with one another through near-field coupling, allowing the transfer of binary information without any current flow. Much of the operation of QCA relies on a clock cycle, which is further divided into phases to control the times at which the information is allowed to flow; each phase dictates whether a cell is to set, switch, hold, or relax. Each QCA cell’s area is determined by the physical spacing of quantum dots within the cell and the distance required to allow effective interaction. At the same time, it ensures that interference is kept at a minimum for QCA technology to scale. Electrons are positioned diagonally to one another and experience reciprocal repulsion as a result of the Coulomb interaction. More specifically, a single cell has to be in one of the two energy states, denoted as *P = +1 (binary 1) and P = -1 (binary 0)*, which are referred to as cell polarization [15]. Additionally, semiconductor QCAs include a four-phase clocking mechanism with a *900-phase* shift to each other in order to perform logical operations and signal propagation. As seen in Fig 1, there are two basic circumstances that dictate electron locations in cells. Additionally, Fig 2 depicts the typical QCA wire. Using regular QCA cells (*90-degree cells*), this QCA wire is produced [16].

**Fig 3:**
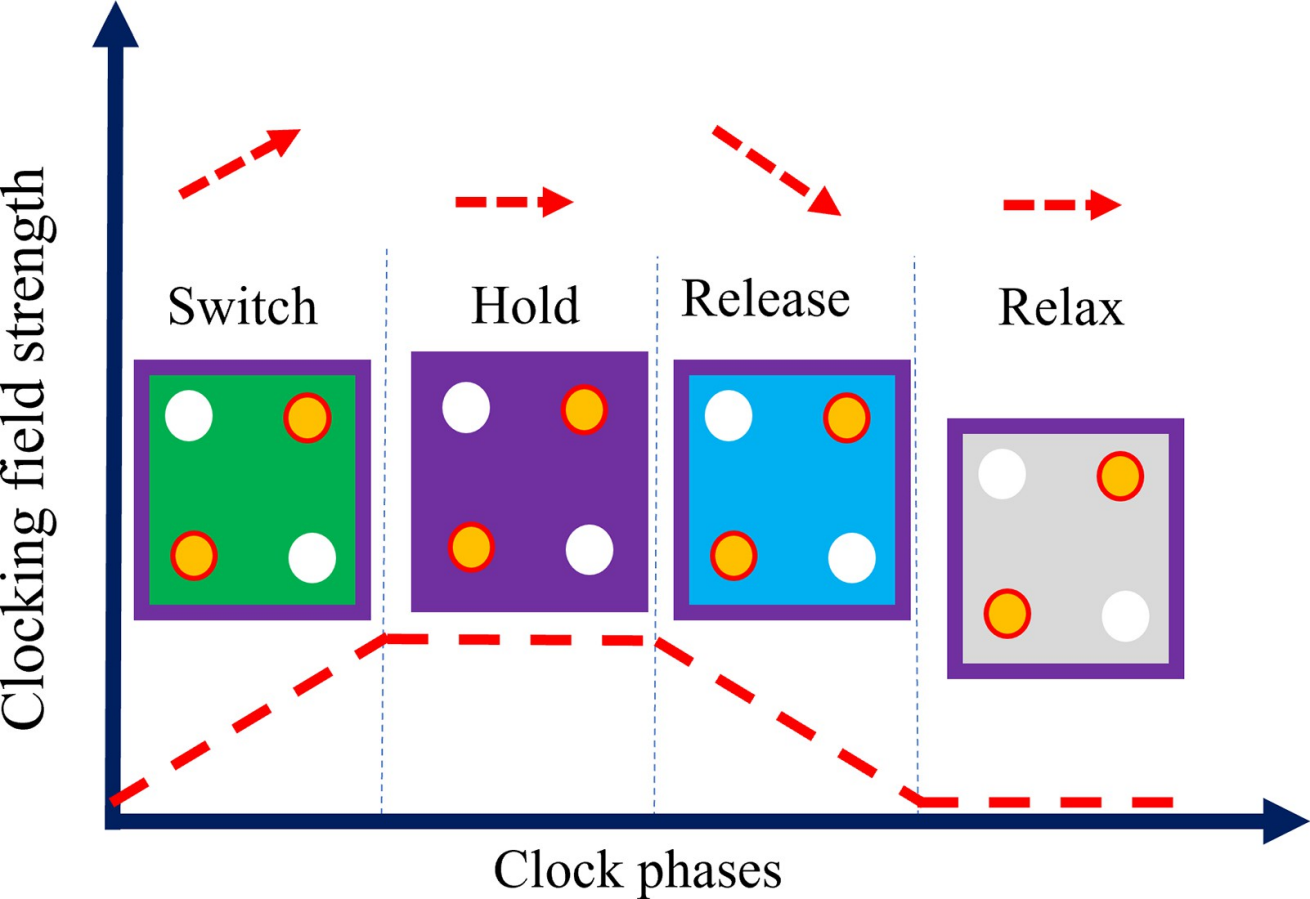
QCA clocking.

A four-phase clocking technique for QCA is shown in Fig 3. The cell is unpolarized in the first phase due to Coulombic interactions with nearby cells. The cell is polarized according to the input drivers by raising the barrier, and after this phase, the barrier is raised sufficiently to stop tunneling. As a result, the cell’s condition is fixed and locked. Take note that this phase is when the changeover occurs. The "*hold*," or second phase, maintains the barrier at a high level. The cell is stable throughout this phase, and information is shared with nearby cells. Barriers progressively drop during the third phase, known as the "*release*," and the cell becomes unstable. At this point, the cell data becomes unpolarized and moves into the Relax phase, meaning it is no longer needed. The cell is unpolarized and the barriers are at their lowest point during this last, fourth phase. The cell is then prepared to go back to the switching phase. QCA differentiates between roles and states of cells in the circuit by color. In this way, it uses blue for input cells, where data is introduced into the system, and yellow stands for output cells where the result comes out. On the other hand, timing control in the QCA is realized through division of the whole circuit into so-called clock zones. Green areas correspond to *Clock 0*, with cells set; purple corresponds to *Clock 1*, with cells actively switching; turquoise corresponds to *Clock 2*, with cells stabilized; and, finally, white corresponds to *Clock 3*, with cells relaxed and ready to be reset or prepared for the next cycle. Color-coded clocks realize parallel and sequential information processing within a QCA system [17, 18].

**Fig 4:**
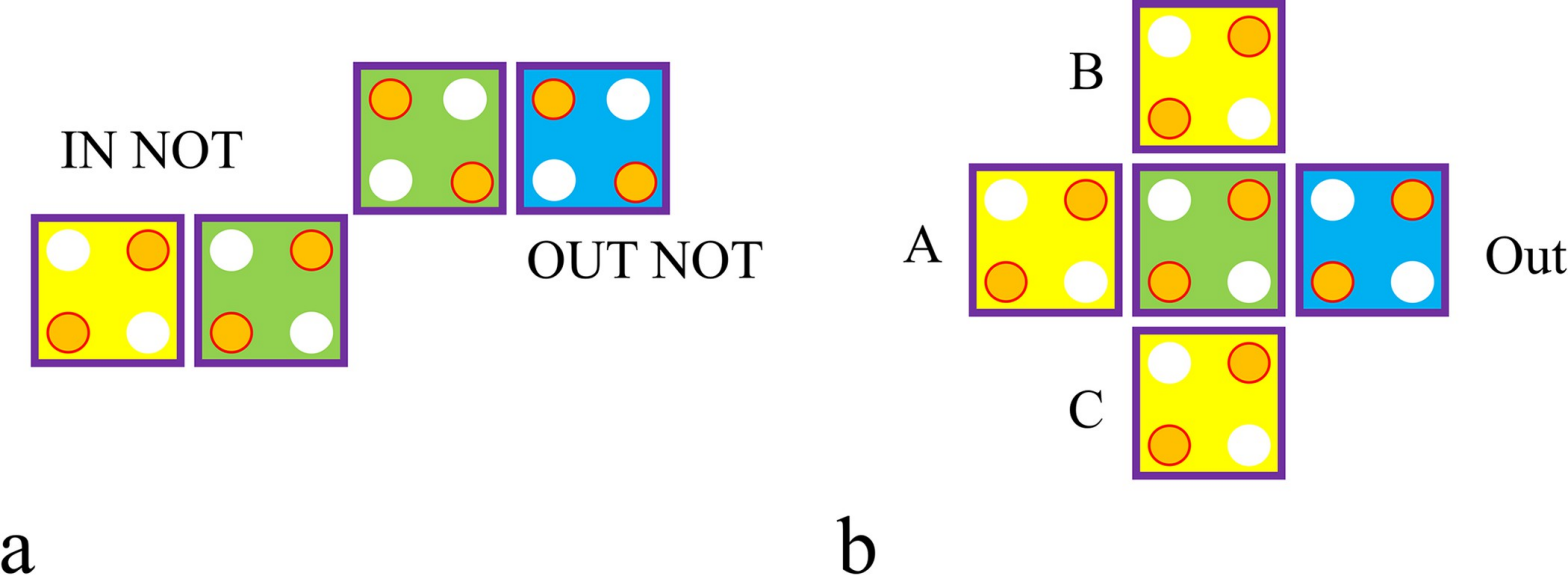
(a) Inverter gate, and (b) Three-input majority gate.

The two basic logic circuits in QCA nanotechnology are the majority gate and the inverter. In QCA nanotechnology, there are several types of inverters, and the majority gate designs are based on the quantity and layout of QCA cells. One may create a basic inverter using a corner-to-corner arrangement. The most basic majority gate has three inputs, although any digital circuit may be created using *2-input AND and OR* operations. Fig 4 shows a corner-to-corner arrangement of an inverter and a three-input majority gate [19, 20].

The importance of approximate computing in this QCA technology cannot be excluded from the above points. It is obvious that approximate computing, as a paradigm, is tolerant of a certain extent of imprecision at the output, so it is essential for optimization in computer operations [10]. Approximate computing in QCA would be a tactical way to balance resource utilization with computational precision whenever speed and energy economy are of essence [9]. QCA technology, realized for its low power, small size, and high speed in processing, benefits greatly from approximate computing. QCA circuits can maintain results sufficient for some applications while reducing energy consumption and raising overall efficiency by the use of approximate methodologies. Other than aligning with the broader trend of energy-efficient computing, an incorporation of approximate computing ideas into QCA opens several new avenues for design and approach experiments. Only proper concepts of approximation in a computation really align with the intrinsic properties of QCA and ensure energy-efficient, high-performance computing at the nanoscale [11].

### 2.2. Related works

In this section, some of the latest articles in the field of approximate FA, FS, FA/S, and RCA have been reviewed.

Jahanshahi Javaran, et al. [21] have presented a unique fault-tolerant five-input majority gate that they claim to be a robust QCA FA. In light of the need of building circuits that can tolerate errors, researchers provide a five-input majority gate with a fault-tolerant feature in QCA technology in this paper. When placing cells in particular places on the surface, researchers evaluate any possible flaws as well. These mistakes include rotation, displacement, deletion, and additional cells. In the first stage, the gate under investigation is exposed to the four failure types outlined before. In the second stage, the correctness of the circuit performance is evaluated using the QCADesigner simulator engine. This five-input majority gate is constructed with *41 cells*.

Four-bit QCA RCA circuits and QCA FA circuits with efficient designs have been presented by Lee, et al. [22]. In QCA, digital arithmetic operations are carried out using adder circuits, while binary information is represented by quantum dots. In digital electronics, a digital circuit called *RCA* is used to add several binary values together. Because of the special characteristics and tenets of QCA technology, the implementation of such adders differs significantly from conventional *CMOS-based* implementations. This is constructed by cascading many FAs together. In this paper, the researchers have implemented the QCA technology in designing a circuit of *4-bit RCA* and an FA. *QCADesigner 2*.*0*.*3* has been used to simulate the suggested designs in a coplanar crossover manner.

In this context, Soyane, et al. [23] have introduced another new reversible gate and another improved design for implementing a half adder, a subtractor, and a *2-bit* multiplier. In fact, a unique *3 × 3* reversible gates with varying functionality for arithmetic and logical operations is proposed in this study. The constant development of *DSP* applications necessitates quicker and more power-efficient multiplier design. One effective solution to the aforementioned issues is reversible logic. The article discusses a basic *2 × 2 multiplier*, a unique gate that has been suggested, and how it may be used to create a half adder-subtractor more effectively than current basic reversible gates. On QCA Designer, the suggested ideas were put into practice.

A suggested quantum-based serial-parallel multilayer (*SPM*) circuit using an efficient nano-scale serial adder has been made by Wu, et al. [24]. In order to improve the overall design and maximize the performance of the multiplier circuit, this research offers a QCA-based SPM circuit. The suggested framework combines effective path planning with a high-performance architecture. Apart from that, the majority gate and *1-bit* serial adder (*BCA*) form the foundation of the suggested QCA-based SPM circuit. The BCA circuit requires 0.5 clock cycles, *0*.*04 μm*^*2*^ in area, and 34 cells. The results indicated that the proposed QCA-based SPM circuit has a delay of *1*.*25 clock cycles*, employs *222* QCA cells, and only takes up *0*.*28 μm*^*2*^ of space. This paper adds to the body of knowledge already available on QCA technology by highlighting its potential to advance VLSI circuit designs through enhanced performance.

Chugh and Singh [2] have finally suggested adder designs for QCA. In order to integrate QCA technology and overcome the size, latency, and cost constraints associated with adder circuits, this research takes advantage of QCA benefits. Researchers presented a FA architecture in QCA that uses a half-adder design with *23* cells in a single layer and no crossover, resulting in a *0*.*25 clock cycle* delay. The designs that were presented were able to achieve a cost efficiency of *64*.*3%* and notable improvements in many design metrics by using the inter-cellular effects that are inherent in QCA technology. This set them apart from conventional logic design methods. Eight- and four-bit ripple carry adders were also constructed using the adder architecture. Simulation designs were tested and utilized by QCADesigner-E.

## 3. Proposed approximate designs

Approximate logic can do intentionally incorrect computations within a certain tolerance range, sacrificing accuracy for higher speed and power economy. This is particularly relevant in areas where perfect precision is not required, like machine learning or multimedia processing. By relaxing the constraints on the requirement for accuracy, approximate logic seeks to deliver flexible and resource-friendly solutions. Traditional Boolean operations are relaxed with approximate logic, ensuring acceptable results lie within pre-defined error bounds. The statement means that as traditional binary logic is deviated from, circuits ideal for energy-efficient computing can be constructed with reduced power and space requirements.

Approximate FAs, based on QCA, extend the notions of approximate logic to the fundamental addition action. These circuits further introduce controlled imprecision to the result and differ from traditional QCA-based FAs. This can be achieved by probabilistic methods or by modifying standard binary addition algorithms. Approximate FAs based on QCA exhibit a resource efficiency–a degree of approximation trade-off in their design. In the same way, approximate FSs based on QCA differ from the corresponding deterministic ones by allowing inexactness in subtraction. These circuits are designed using the QCA framework, which may be simplified architectures, probabilistic methodologies, or approximate carry-save or borrow-save techniques. This degree of approximation can be further fine-tuned to trade off with the exactness of computation insofar as the application requires. Approximate *FA*, *FS*, *FA/S and RCA* can have different degrees of approximation according to the needs of the application in question, balancing resource efficiency with computational precision.

**Fig 5:**
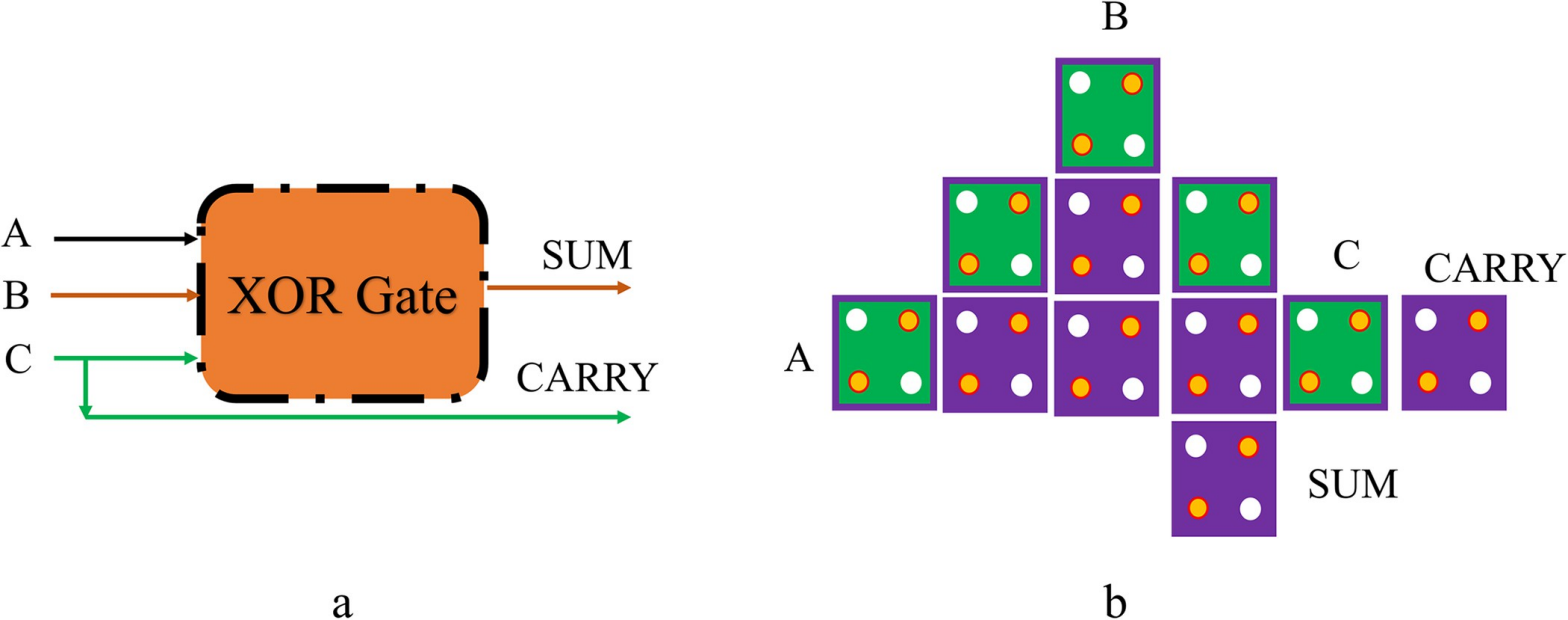
The proposed approximate FA in QCA: (a) Schematic, and (b) Layout.

**Fig 6:**
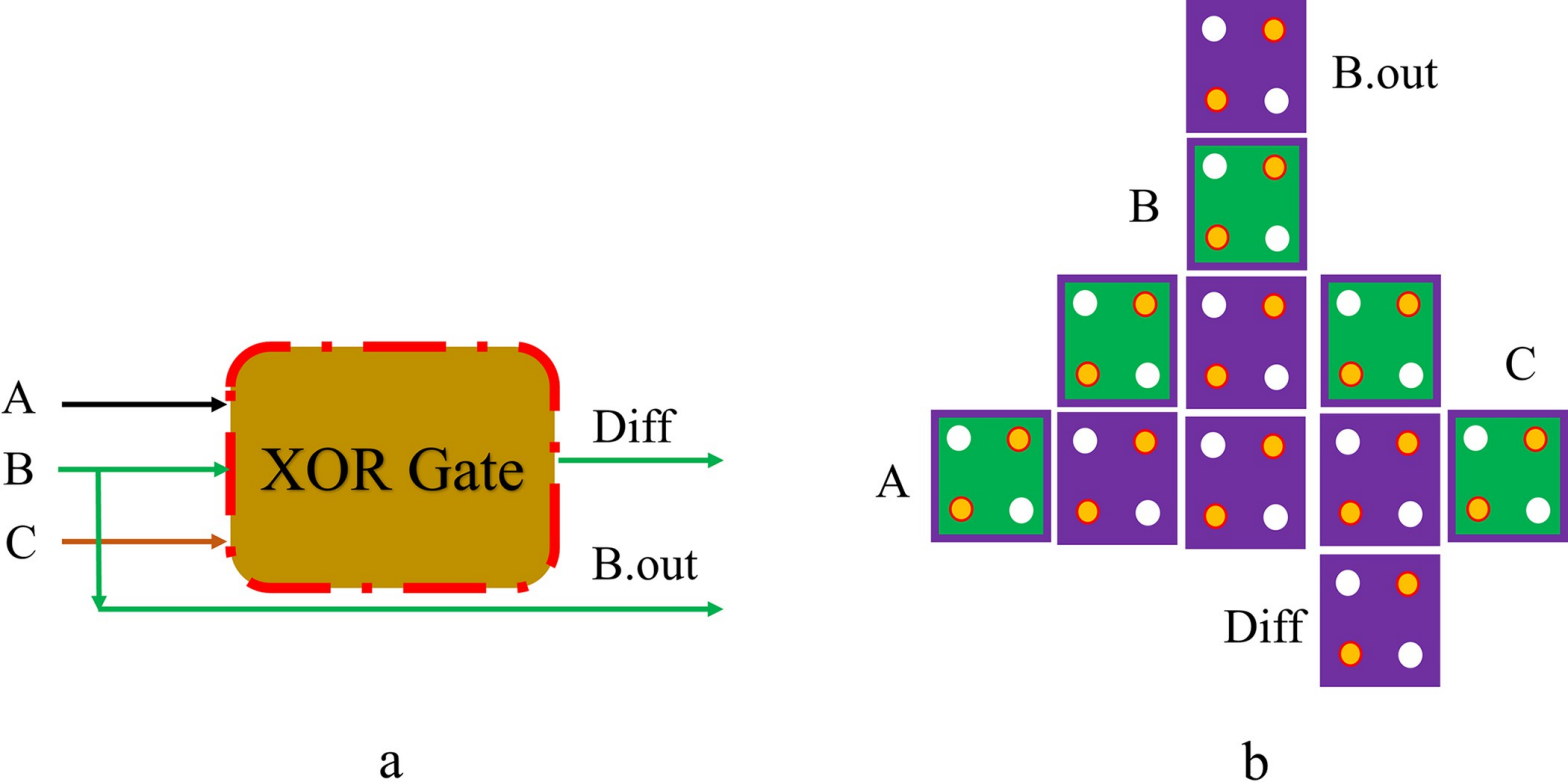
The proposed approximate FS in QCA: (a) Schematic, and (b) Layout.

An approximate FA is a combinational logic circuit with three inputs, producing a SUM and a CARRY bit. Fig 5(A) illustrates the design layout of an Approximate FA in QCA, implemented using a single XOR gate. In contrast, Fig 5(B) depicts the proposed schematic for an Approximate FA in QCA. With a circuit size of *0*.*01 μm*^*2*^, the total cells used to design are only *11*, giving the output after *0*.*5* clock phases. Fig 6(A) also depicts the block schematic of an estimated FS. The new design for the given approximate FS is shown in Fig 6(B). Only two clock phases and eleven QCA cells are required to implement the approximate FS, which is elaborated. These circuits further inject controlled imprecision into the results, making them different from traditional QCA-based FA, FS, and FA/S circuits. Probabilistic approaches or modifications in some standard binary addition algorithms can be used to achieve the required degree of approximation. A critical trade-off between the degree of approximation and resource efficiency is always guaranteed in the QCA-based approximate FA, FS, and FA/S circuits design. The approximate FS suggested above requires an estimated *0*.*01 μm*^*2*^ of space. The above optimum FS is also demonstrated with the help of the XOR gate. The input signals, which are applied to the circuit, are A, B, and C. There exist two outputs with a value of one for every input state. As the XOR gate produces Diff and the input B produces B.out, the above circuit produces both Diff and B.out. Table 1 also includes an approximate truth table for the FA and FS circuits. The approximate FA circuit consists of a single XOR gate, correctly calculating the total output for every possible input but introducing a *25%* error to the carry output, corresponding to two of eight possible carry outputs. The FS approximation circuit also yields a correct Diff output, but the B.out output is wrong *25%* of the time in *2* of the *8* cases.

**Table 1:** The FA, FS, and FA/S truth table.

A	B	C	Diff	B.out	SUM	CARRY
0	0	0	0	0	0	0
0	0	1	1	1	1	0
0	1	0	1	1	1	0
0	1	1	0	1	0	1
1	0	0	1	0	1	0
1	0	1	0	0	0	1
1	1	0	0	0	0	1
1	1	1	1	1	1	1

**Fig 7:**
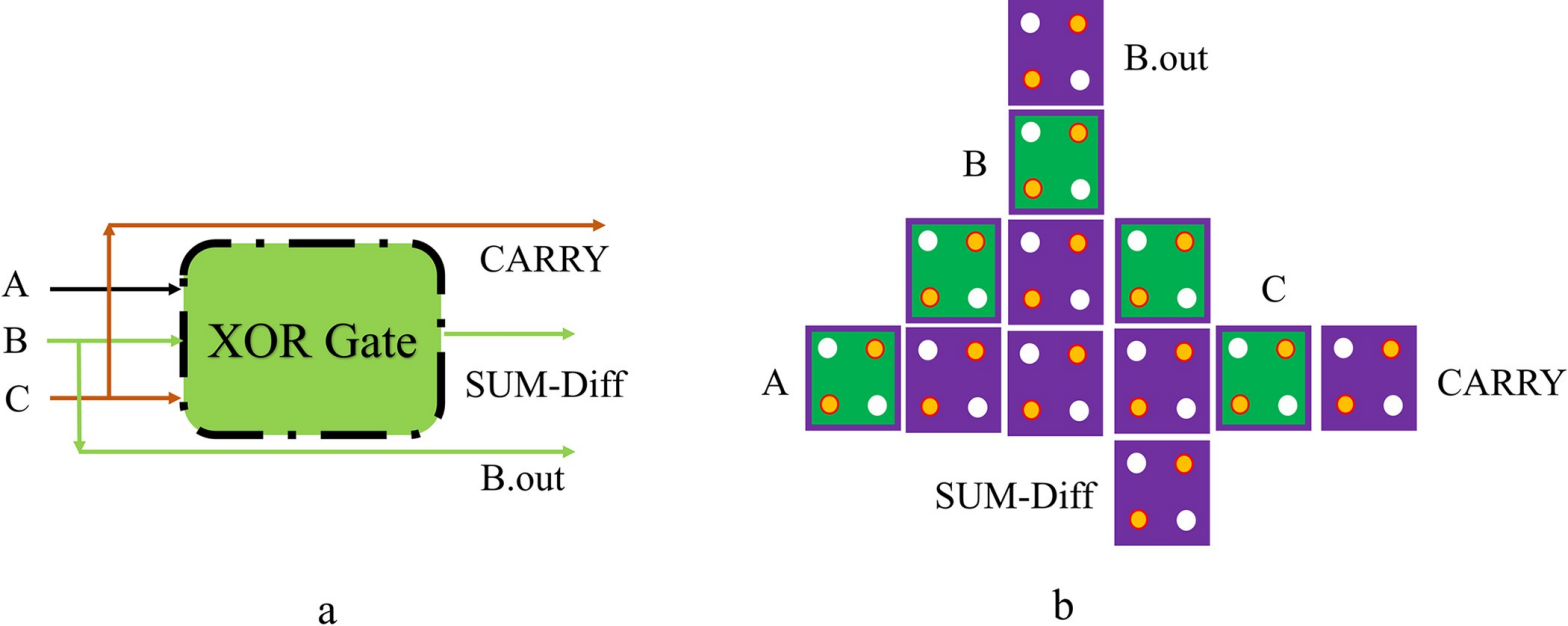
The proposed approximate FA/S in QCA: (a) Schematic, and (b) Layout.

Furthermore, approximate FA/S remains among the prime elements that form digital computational circuits. The approximate FA/S design developed has a small number of cells, only *12*, and a small circuit area of *0*.*01 μm*^*2*^. The output from the design provided is produced after 0.5 phases of a clock. Fig 7(A) provides a block schematic of an approximate FA/S, and Fig 7(B) provides the QCA design for the approximate FA/S. Several QCA-based circuits for FA, FS, and FA/S have been proposed so far, forming the basis of Section 2. Therefore, these designs represent continuous research and development in the QCA domain to increase the efficiency and applicability of digital circuits. These circuits help build energy-efficient digital systems and allow controlled imprecision in computations to fulfill the myriad requirements of modern-day applications. This fact demonstrates the high dynamics in conducting research in the field of QCA with the design of new methodologies and optimization strategies. At the same time, it also poses opportunities to integrate approximate computing in conventional digital circuits. The approximate FA/S circuit delivers the results in *0*.*5* clock phases and generates the *25%* error in the CARRY/B.out output with *2* out of *8* cases mistaken. But it gives correct sum/difference outputs.

The operation of the FA, FS, and FA/S outputs, which can be represented by Eqs (1), (2), and (3):

CARRY=(A.B)+(A.C)+(B.C) (1)

B.out=(A’.B)+(A’.C)+(B.C) (2)

SUMandDiff=A⊕B⊕C (3)

The approximate FA, FS, and FA/S outputs, as well as the approximate Eqs (4), (5), and (6), can be expressed as follows:

CARRY=C (4)

B.out=B (5)

SUMandDiff=A⊕B⊕C (6)

**Fig 8:**
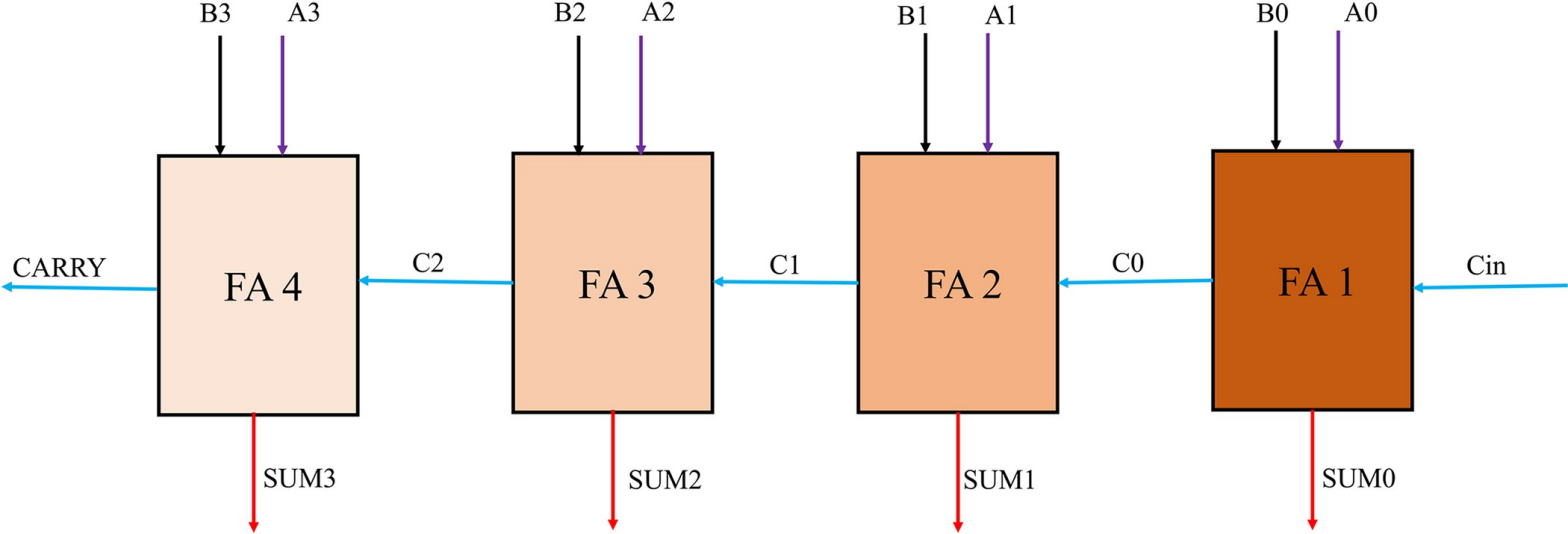
The proposed schematic for approximate RCA.

**Fig 9:**
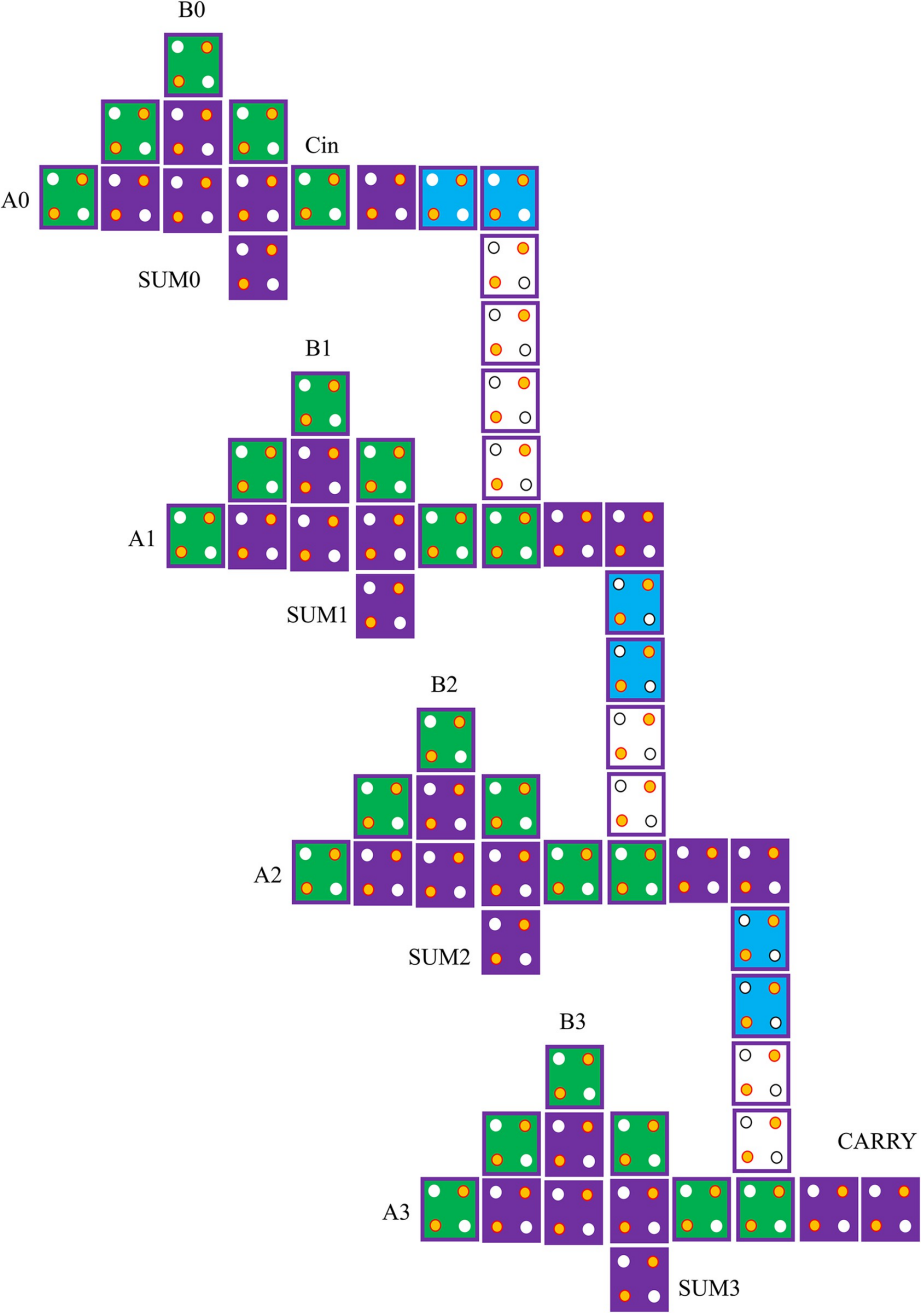
The proposed layout for approximate RCA in QCA technology.

In actuality, RCA circuits and approximation FAs are basic building blocks of digital arithmetic and logic circuits. The architecture and implementation of approximate RCA are straightforward, but because each full adder must wait for the computed carry bit from the preceding one, the system operates slowly. Fig 8 depicts the layout of the suggested four-bit approximation RCA architecture. Furthermore, the suggested *four-bit* approximation RCA with four FAs that was created using QCA technology is shown in Fig 9. We leverage single layer technology and explicit interactions between QCA-based cells to develop the approximation RCA QCA-based circuit. Actually, mono-layered circuits are possible in this architecture because of the coplanar crossing. All of the inputs and outputs of this circuit are situated in a single layer due to its one-layer architecture. There are five outputs (*SUM0-SUM3*, *CARRY*) and nine inputs (*A0-A3*, *B0-B3*, *Cin*) in this layer. The outputs in this design are easily accessible since they are not encircled by other cells. Put otherwise, this system transmits the output signal without the need for a cable. As a result, feeding the outputs to the input of another QCA-based circuit is simple. With *64* cells and a *0*.*11 μm*^*2*^ size, the suggested four-bit approximation RCA in QCA technology has a *3*.*5 clock cycle* delay.

We need to get the total error rate (*ER*) for a *4-bit* RCA made up of four approximation adders, each of which has a *25%* error rate for the carry output. Given the *0*.*25* error rate, the likelihood of a single carry output being right is *0*.*75*. The *4-bit* RCA’s four carry outputs have the following likelihood of being correct:

$$P(All\;correct)=0.75^{4}=0.3164$$ (7)

As a result, the likelihood that at least one carry output is incorrect is:

ER=P(Atleastonewrong)=1–0.3164=0.6836 (8)

Thus, the overall error rate for the carry output in the 4-bit RCA is approximately *68*.*36%*.

The discrepancy between the actual and predicted carry output numbers is known as the Error Distance (*ED*) [25]. We must take into account each stage’s influence because there are four phases. The total of the mistakes supplied by each bit location determines the maximum error distance that can exist in a binary system. The weight of the bit location is the probable error distance for each incorrect carry.


$$ED=2^{0}+2^{1}+2^{2}+2^{3}=1+2+4+8=15$$ (9)

Also, the average error distance over all potential mistakes is known as the Mean Error Distance (*MED*). With a *68*.*36%* error rate, the *MED* may be computed as follows:

MED=ER×ED=0.6836×15=10.254 (10)

The ratio of the MED to the maximum error distance is known as the mean relative error distance (*MRED*) as follow:

$$MRED=\frac{MED}{{ED}_{max}}=\frac{10.254}{15}=0.6836$$ (11)

Lastly, the average number of incorrect carry outputs each stage may be used to compute the Mean Error (*μ*). Considering the 68.36% error rate for four stages:

μ=ER×4=0.6836×4=2.7344 (12)

## 4. Simulation tool, parameters, and results

The modeling of the FA, FS, and FA/S circuits that have been optimized for area and cell in QCA technology is shown in this part. The coherence vector simulation models and the QCADesigner 2.0.3 tool environment were utilized to complete the layout design phases. Throughout the design phase, the temperature must be maintained at *2 Kelvin*, and the Euler approximate approach is applied. The clock’s low value is *3*.*8e-023*, and its maximum value is *9*.*8e-022 Joule*. Using the fewest QCA cells possible, all configurations for the FA, FS, and FA/S circuits are suggested. The FA, FS, and FA/S circuit architectures are being presented to evaluate the quantum cost for various levels of complexity and occupied space. Furthermore, no prior research has been done on quantum cost [26]. Table 2 describes how simulation engines are set up in the QCADesigner tool to be "*Bistable Approximation*" and "*Coherence Vector*" kinds [26, 27].

**Table 2:** QCADesigner parameters for bistable approximation engine and coherence vector engine values.

Parameter	Bistable approximation engine Value	Coherence Vector engine Value
Cell size	18×18 nm^2^	18×18 nm^2^
Radius of effect	65 nm	80 nm
Relative permittivity	12.9000000	12.9000000
Clock high	9.8e−22J	9.8e−22J
Clock low	3.8e−23J	3.8e−23J
Clock amplitude factor	2.000000	2.000000
Clock shift	0.000000e+000	0.000000e+000
Layer separation	11.5000 nm	11.5000 nm
Maximum iterations per sample	100	-
Number of samples	12800	-
Convergence tolerance	0.001000	-

**Fig 10:**
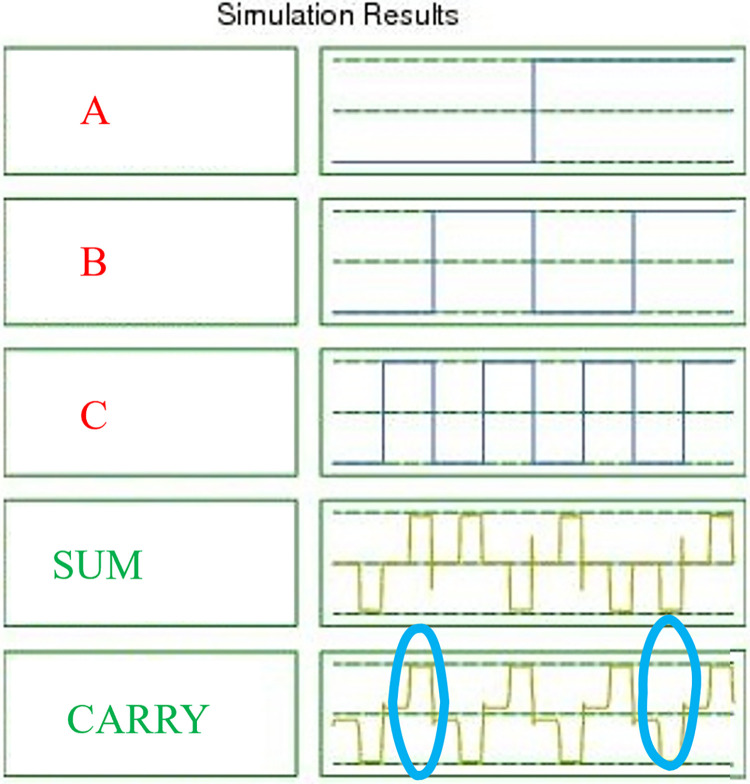
The simulation results for the proposed approximate FA.

The simulation results of the suggested approximate FA employing QCA technology are shown in Fig 10. The inputs *A*, *B*, *and C* that were extensively examined are shown in this figure. The results validate the design by matching the theoretical approximate FA values in QCA technology. With the exception of those indicated by circles in *CARRY*. It should be noted that *SUM* is *100 percent* correct. It should be noticed that the *CARRY* output produces false results in two particular circumstances. Additionally, we have used this software to apply the input in every scenario that might result in output via an approximation RCA circuit in QCA technology.

**Fig 11:**
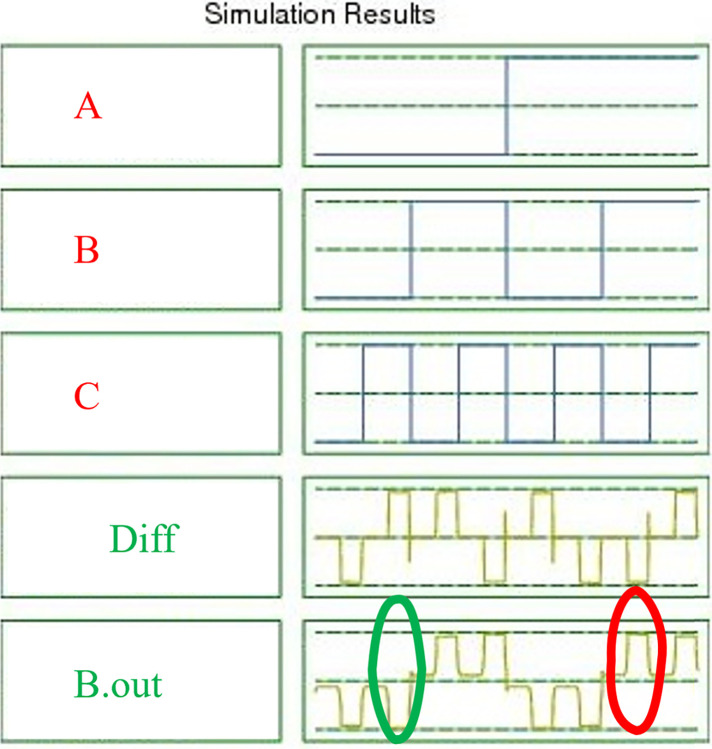
The simulation results for the proposed approximate FS.

Fig 11 displays the simulation results of the proposed approximate FS using QCA technology. The well-researched inputs *A*, *B*, *and C* are represented in this figure. The concept’s validity is demonstrated by the findings, which align with the theoretical approximate FS values in QCA technology. With the exception of those in *B*.*out*, which are shown by circles. It should be mentioned that Diff is *100%* true. It should be noted that the output *B*.*out* yields inaccurate results in two cases.

**Fig 12:**
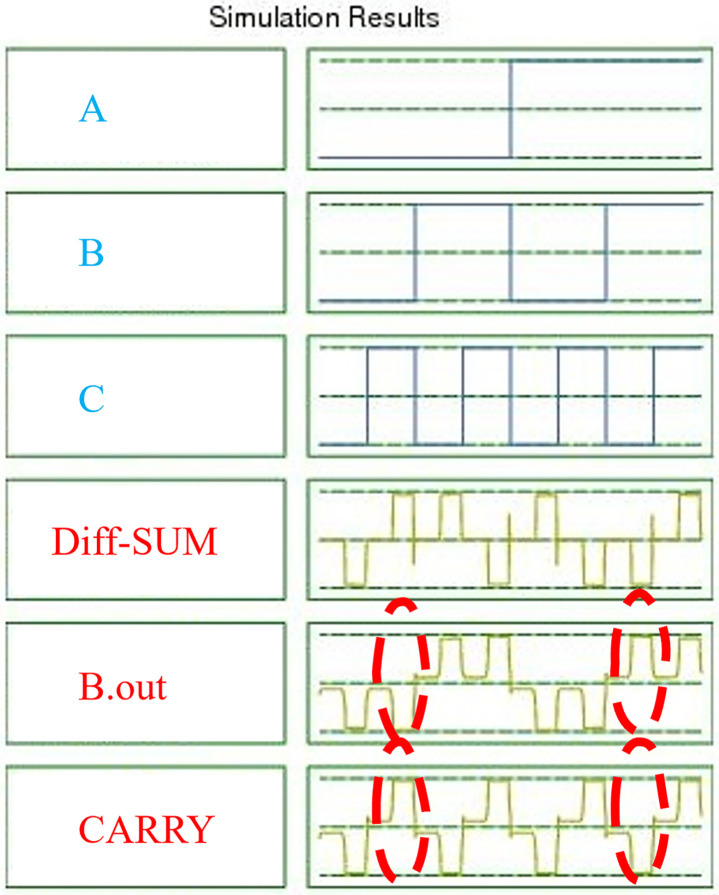
The simulation results for the proposed approximate FA/S.

Fig 12 illustrates the simulation results of the presented approximate FA/S using QCA technology. The figure shows the precisely examined inputs A, B and C. These results confirm the conceptual validity because they are very close to the theoretically predicted values for approximate FA/S in QCA technology. Noticeably, the fact that there is no difference in Diff and SUM means the accuracy is retained. Circles show where B.out and CARRY are different. It should, therefore, be noted that the differences as seen do not alter the accuracy of Diff and SUM.

Table 3 provides a detailed comparison to indicate the improvement of various QCA-based approximate FA, approximate FS, approximate FA/S, and approximate RCA that were recently proposed. The proportion of improvement in cell count of the suggested designs over the best designs is shown in the table’s last column. The FA design is *21%* better than the previous best design, while the FS design is *21%* better than the previous best design, based on the improvement percentages that were offered. The number of cells in the proposed approximate *4-bit* RCA is reduced by *43%* compared to the best previous design. The area occupied, the number of cells, latency, and quantum cost (*Area × Latancy*^*2*^) are significant factors in assessing the feasibility and efficiency of digital circuit designs. Results have shown that the proposed QCA-based designs outperform all previous predecessors in cell number, quantum cost, and complexity. This increase means a more streamlined and compact structure and is intended to mean a more efficient usage of the resources.

**Table 3:** Comparison among the QCA-based proposed approximate designs.

Designs		Area (*μm*^*2*^)	Cells	Latency (clock phases)	Cost	Percentage improvement in terms of cell count
**FA**	**Proposed approximate FA**	**0.01**	**11**	**0.5**	**0.0025**	**-**
	Aravinth and Marcilin [28]	0.04	35	0.75	0.0225	69%
	Moaiyeri, et al. [8]	0.04	33	0.75	0.0225	67%
	Das and Singh [29]	0.02	32	0.50	0.0050	66%
	Zhang, et al. [10]	0.02	14	0.50	0.0050	21%
	Bahar and Wahid [11]	0.01	17	0.50	0.0025	35%
	Ahmadpour, et al. [30]	0.01	20	0.25	0.0006	45%
	Seyedi and Navimipour [13]	0.01	22	0.75	0.0056	50%
	Sarmadi, et al. [31]	0.04	30	1.00	0.0040	63%
	Sayedsalehi, et al. [32]	0.02	31	0.75	0.0112	65%
	Sayedsalehi, et al. [32]	0.02	33	0.75	0.0112	67%
	Gassoumi, et al. [33]	0.01	19	0.50	0.0025	42%
**FS**	**Proposed approximate FS**	**0.01**	**11**	**0.50**	**0.0025**	**-**
	Bahar, et al. [34]	0.03	32	0.50	0.0072	66%
	Gassoumi, et al. [33]	0.01	20	0.50	0.0025	45%
	Zhang, et al. [10]	0.02	14	0.50	0.0050	21%
	Bahar and Wahid [11]	0.04	44	0.75	0.0225	75%
	Das and Singh [29]	0.02	32	0.50	0.0050	66%
**FA/S**	**Proposed approximate FA/S**	**0.01**	**12**	**0.50**	**0.0025**	**-**
**RCA**	**Proposed design approximate RCA**	**0.11**	**64**	**3.50**	**1.3475**	**-**
	Seyedi and Pourghebleh [6]	0.17	125	2.75	1.2856	49%
	Chan, et al. [35]	2.50	1246	5.75	82.656	95%
	Safoev and Jeon [36]	0.11	184	1.25	0.1718	65%
	Roshany and Rezai [37]	0.17	125	1.25	0.2656	49%
	Seyedi, et al. [38]	0.13	112	1.00	0.1300	43%
	Chudasama, et al. [39]	18.44	13533	10.75	277.02	99%
	Balali and Rezai [40]	0.31	209	1.25	0.4843	70%
	Rashidi and Rezai [41]	0.14	175	1.00	0.1400	63%
	Abedi, et al. [42]	0.21	262	1.75	0.6431	76%
	Mohammadi, et al. [43]	0.24	237	1.50	0.5400	73%

Further, the proposed designs achieve the same latency as some of the most popular previous solutions, thus proving that a fair balance in the trade-off between computational complexity and speed is required. The lower circuit complexity will mean a simplified and efficient design, lessening the usually cumbersome complexity associated with traditional QCA techniques. Although this new four-bit approximation RCA in QCA technology is delayed by *3*.*5 clock cycles*, this design has several advantages concerning access to inputs and outputs. Moreover, Table 3 assigns a low number of cells and an area of implementation compared to others; all of these make the proposed solution more efficient and practical. As mentioned earlier the proposed design 4-bit RCA contains *43%* fewer cells than the best previous design. Such a low number of cells will agree very well with the trend of efficient and sustainable circuit design and, hence, will contribute to a compact structure and predict better resource utilization. This makes the economic designs recommended from these results economically feasible for their implementation and by related low-cost reachable for real-world applications. One major factor in the diffusion of new technologies is their affordability. It is also easier to integrate these designs into larger systems and allow them to interact with other elements more transparently due to the ease by which inputs and outputs can be accessed.

## 5. Conclusion and future works

Quantum cellular automata (*QCA*) technology was selected as one of the best options due to its high density and low power consumption; it is also faster than *CMOS* technology. With the newness of QCA technology, several of its fundamental problems have yet to be explored and understudied. One of the significant issues in this regard is the approximate computing problem. Approximate computing has lately represented one of the techniques bringing a shift in paradigm to nanoscale technologies that showed use in processing within the computational process and offered a low-power design approach tailored toward arithmetic circuits. The adder circuit as a whole is of the essence to every arithmetic logic unit operation, including division, multiplication, and subtraction in QCA technology. As a result, approximate full adder (*FA*) and approximate full subtractor (*FS*), approximate full adder/subtractor (*FA/S*) *and* approximate ripple carry adder *(RCA)* with *XOR* logic became very popular and have been considered lately for the most required sections of QCA-based digital arithmetic circuits. This paper proposes three new and efficient QCA circuits with an approximate *FA*, an approximate *FS*, and an approximate *FA/S* based on single-layer *XOR* logic. These designs have inputs on one side and outputs on the other, making them easily accessible without being enclosed by any other cells and making them more efficient circuit designs. Regarding the area, both FA and FS designs achieved an area of approximately *0*.*01 μm*^*2*^, implemented in just *11* cells, with a delay of *0*.*5 clock phases* in detail. Comparably, the approximate FA/S designs exhibited only *12* cells to be implemented, an area of *0*.*01 μm*^*2*^, and a delay of *0*.*5 clock phase*. The efficiency of these designs has already been proved through functional verification using the QCADesigner tool. The simulation results will confirm how much quicker and smaller these proposed designs are compared to the old ones and how much better they function. The FA design beat the previous best design by *21%*, while the FS design beat the previous best design by *21%*, according to the improvement percentages that were supplied. The *4-bit* RCA in the proposed design has *43%* fewer cells compared to the best previous design. These designs are perfect and practical; hence, notable advancements in digital circuits based on QCAs. These ideal designs significantly advance and open new pages when developing QCA digital circuits. Further future research and development in this line are expected to open for these concepts a door for more intricate and complex applications of QCA technology in digital circuits.
